# Delphi study to elicit expert consensus around decision-making in the treatment of Friedreich ataxia

**DOI:** 10.3389/fneur.2025.1669059

**Published:** 2025-10-24

**Authors:** Sheng Han Kuo, Cuixia Tian, James McKay, Sarah M. England, Monica Simon, Marlon Graf, Iris P. Brewer, Natalie Land, Jacquelyn W. Chou, Richard Lawson

**Affiliations:** ^1^Columbia University Medical Center, New York, NY, United States; ^2^Cincinnati Children's Hospital Medical Center, College of Medicine, University of Cincinnati, Cincinnati, OH, United States; ^3^Biogen Inc., Cambridge, MA, United States; ^4^Precision AQ, Bethesda, MD, United States

**Keywords:** Friedreich ataxia, Delphi, expert consensus, diagnosis, treatment decision-making, clinical outcomes assessment scales

## Abstract

**Introduction:**

Friedreich ataxia (FA) is a rare neurological disease. This study aimed to understand current FA management and treatment practices among neurologists.

**Methods:**

An online, modified Delphi study consisting of 34 United States (US)-based neurologists with varying levels of FA experience was conducted. The Delphi questionnaire consisted of likelihood, ranking, and parameter estimation questions related to FA decision-making practices. Data collection occurred in 3 sequential rounds: completion of Delphi questionnaire, webinar discussion, and re-completion of the questionnaire. Consensus was reached in Round 3 if the inter-quartile range [IQR] was ≤25 around the median group response [MED] (scaled 0–100) or if ≥75% of panelists ranked an option in the top-2. Results were analyzed for the full panel and separately for experienced FA-treaters.

**Results:**

Panelists strongly agreed overlapping symptoms with other ataxic or neurologic conditions is a key diagnostic challenge (MED = 90, IQR = 1.7) with misdiagnosis being the most important driver in delayed FA diagnoses (MR = 1.7, 65% top-2). General neurological exams were the most frequently used tool to assess FA disease progression (MED = 100, IQR = 0) whereas panelists were largely unfamiliar with any of the clinical outcome assessment scales provided (MED range: 5–10, IQR range: 20–23). Experienced FA-treaters’ responses were largely consistent with the full panel; however, some differences were observed.

**Discussion:**

Consensus was reached on a portion of questions regarding FA diagnosis and assessment, perhaps due to the rarity of disease and panelists’ varying FA experience. To improve and standardize management of FA, it is important to establish best practices and educate potential FA treaters as new therapies emerge.

## 1 Introduction

Friedreich ataxia (FA) is a rare genetic neurological disease. In the United States (US), FA was estimated to affect 1 in 50,000 people (1). FA is caused by pathogenic trinucleotide repeat expansions in the *FXN* gene, which is responsible for the production of the frataxin protein. These pathogenic variants have downstream implications through a disruption of iron metabolism in the mitochondria, leading to progressive neurodegeneration (2).

FA impacts the central and peripheral nervous system, and while the clinical phenotype is broad, the disease typically involves gait and limb ataxia, bulbar impairment, and occasionally hearing loss, vision impairment, and mild sense of smell dysfunction (2). FA is also strongly associated with cardiomyopathy, atrial fibrillation, and diabetes mellitus (2, 3). The average age of onset for FA is between 10 and 15 years of age (4). The disease progression may lead to loss of ambulation and use of a wheelchair approximately 15–20 years after the initial onset (3). Mortality data on FA is limited, but it is well documented that the pathophysiological severity of this disease shortens life expectancy (5).

Current management of FA requires a multidisciplinary approach focused on symptom management (6, 7). People living with FA undergo routine monitoring and screening for neurologic, cardiac, endocrine, ophthalmologic, orthopedic, and mental health (2, 6). Genetic counseling is recommended to discuss the possibility of inheritance and health implications that require support from family members. Physiotherapy and speech therapy are implemented to help maintain balance and hand dexterity, as well as manage difficulties with speaking and swallowing (2, 6–8).

However, there are challenges with the assessment of FA disease severity and progression. Several validated clinical outcomes assessment scales (COAS) for FA have been developed, including the Brief Ataxia Rating Scale (BARS), International Cooperative Ataxia Rating Scale (ICARS), Scale for the Assessment and Rating of Ataxia (SARA), and modified Friedreich Ataxia Rating Scale (mFARS) (9), but it is unknown whether physicians are familiar with these COAS and use them in real-world practice. While commonly used in clinical trial settings, COAS might be too complex or resource-intensive to administer in the real-world setting, highlighting the need to leverage assessment tools in clinical trial settings that are easy to adopt in routine clinical practice.

To better understand management practices in FA outside of clinical trial settings as new therapies emerge, we conducted a modified Delphi study to characterize decision-making processes and formalize consensus regarding key factors neurologists in the US consider when making treatment recommendations for patients with FA. The specific objectives of this study were to obtain consensus around decision-making practices for neurologists in the real-world treatment of patients with FA, to gain insights into the COAS used by neurologists as well as feasibility and pragmatic hurdles to implementing COAS in real-world clinical practice, and to understand the key challenges and unmet needs neurologists encounter in the treatment of patients with FA.

## 2 Methods

### 2.1 Study design

Building on the RAND/UCLA Appropriateness Method (RAM) (10–12), this study consisted of an online modified three-round Delphi process conducted between May and September 2024. The Delphi method is a structured and scientifically rigorous approach for eliciting expert opinions and reducing the variance in responses through an iterative process (13). This approach can be used to provide ratings and quantification of information with pronounced uncertainty (14, 15). The Delphi questionnaire utilized in this study was developed based on findings from existing literature on management and treatment of FA and input from two FA clinical experts. In Round 1, participants were asked to complete the questionnaire to provide feedback on various aspects of decision-making processes for neurologists who may treat a patient with FA. During Round 2, participants joined one of a series of webinars where they were shown aggregate answers to Round 1 and asked follow-up questions vetted by clinical experts to provide feedback on their responses. Lastly, in Round 3, the original questionnaire was administered to all panel participants, along with their Round 1 responses, to discern whether the group discussion and aggregated results in Round 2 altered their initial Round 1 assessments. Advarra Institutional Review Board (IRB) exempted this study from IRB oversight (Pro00079057). All participants provided informed consent prior to participation.

### 2.2 Panelist selection

We aimed to assemble a panel comprised of US-based neurologists with varying levels of experience with FA to gather diverse insights from clinicians who may encounter and treat patients with FA in their practice. For the purposes of this study, a physician’s level of experience was determined by the number of FA patients seen, whereby those with substantial/recent experience had treated ≥1 patient with FA in the past year, those with some experience had treated ≥1 patient with FA ever, but no patients in the past year, and those with no experience had never treated a patient with FA. The target panel sample size was 30 to 45 neurologists, with 10 to 15 participants from each experience group, in line with recent Delphi studies (16, 17) and sufficient to provide diversity of expertise and opinions, as well as the ability to confirm and validate shared views across the full sample and by experience subgroup.

Potential participants were identified through a third-party survey vendor’s (Sago) proprietary panel of physicians. All recruitment and data collection occurred in a double-blind manner, meaning the study sponsor and research team did not know the identity of the panelists and panelists did not know the identity of the study sponsor. To maintain the confidentiality and anonymity of the panelists throughout the study, all data associated with a participant was labeled and tracked with a unique identification number only. All panelists were required to be board-certified or specialized in neurology with at least 5 years of clinical practice experience. The panel included pediatric and adult neurologists who have spent at least 50% of their time providing direct patient care or at least 25% of time/effort for providers in the substantial/recent experience subgroup. Further, panelists were required to be age 18 or older, proficient in English, and willing and able to participate in all three web-based rounds of the Delphi process.

### 2.3 Questionnaire

The Delphi questionnaire consisted of three sections with a total of 25 questions designed to elicit ratings, rankings, and uncertainty estimates related to decision-making practices for patients with FA in real-world settings (1): current approaches for identification, diagnosis, management, assessment, and treatment of patients with FA (8 questions) (2), key challenges and unmet needs healthcare providers face in diagnosing and treating patients with FA (6 questions), and (3) familiarity and use of COAS, specifically the BARS, ICARS, mFARS, and SARA, to monitor and assess FA disease activity (11 questions). The questionnaire included 14 likelihood questions, some of which contained multiple items resulting in a total of 86 likelihood estimates. The remaining questions consisted of nine ranking questions and two parameter estimation questions.

For likelihood questions, panelists were asked to estimate the likelihood of a particular event occurring, on a scale of 0 to 100 (where “0” is highly unlikely and “100” is highly likely). For ranking questions, panelists were asked to rank the relative importance of measures or factors on a given outcome. For all ranking questions, panelists were provided with a fixed number of options to choose from. For parameter estimation, panelists were asked to estimate specific values (e.g., time to complete a COAS) within a given range. Wherever necessary, definitions of key concepts were provided to ensure consistency of questionnaire interpretation. For example, a brief overview of each of the COAS and links to each scale were provided at the beginning of that survey section.

### 2.4 Statistical analysis

Descriptive analyses were conducted to summarize participants’ characteristics, including relevant demographic characteristics and characteristics of the participants’ collective expertise in neurology and experience with FA. Only panelists who completed all three rounds of the Delphi process were included in the final analysis.

Final responses submitted during Round 3 were utilized to define areas of consensus and disagreement. Likelihood questions were summarized using the median and inter-quartile range (IQR). Consensus for likelihood questions was considered to be present if the IQR was ≤25, whereas large IQRs ≥75 indicated disagreement (18). The level of likelihood was assessed based on the median, where a median ≥75 indicated high likelihood and a median ≤25 indicated low likelihood. Ranking questions were summarized using mean ranks and the percentage of panelists ranking an item as one of their top two options. An item was considered influential with consensus if ≥75% of panelists indicated the option in the top two ranks whereas an item was considered non-influential with consensus if ≤25% of panelists ranked an option in the top two (19–22). Higher mean ranks indicated higher levels of importance, while lower means indicated lower levels of importance. Parameter estimation questions were summarized using the median and IQR, however, no formal definitions of consensus were applied to these items. Results are reported for the overall sample and differences between the overall and experienced FA-treaters samples were assessed.

## 3 Results

### 3.1 Participant characteristics

Of the 133 potential participants who responded to the study invitation, 80 met the study eligibility criteria, 38 agreed to participate, and 34 completed all three rounds. The final panel was comprised of 13 neurologists with substantial/recent experience treating patients with FA, 12 neurologists with some experience with FA, and 9 neurologists with no prior exposure to FA in their clinical practice. Overall, neurologists had a mean age of 56 years, the majority were male (79%), White (62%), and spent over 15 years practicing neurology (73%). Neurologists’ practice setting was largely in university clinics (38%) or private practices (29%), and practice location was generally in suburbs of large cities (44%) or major metropolitan areas (35%). Table 1 presents the panelists’ demographic information and background expertise. Panelists reached consensus on 24 (28%) of the 86 likelihood estimates provided (Table 2) and 28 (76%) of options they were asked to rank (Table 3).

**Table 1:** Participant background characteristics.

	Full panel (*n* = 34)	Substantial/recent experience (*n* = 13)	Some experience (*n* = 12)	No experience (*n* = 9)
Age, mean (SD)	56 (8.8)	55 (7.3)	57 (9.1)	56 (9.6)
Gender, *n* (%)				
Female	7 (21)	3 (23)	3 (25)	1 (11)
Male	27 (79)	10 (77)	9 (75)	8 (89)
Practice location, *n* (%)				
Major metropolitan area	12 (35)	5 (38)	4 (33)	3 (33)
Urban area	3 (9)	1 (8)	1 (8)	1 (11)
Suburb of a large city	15 (44)	6 (46)	5 (42)	4 (44)
Small city	2 (6)	1 (8)	0 (0)	1 (11)
Rural or small town	2 (6)	0 (0)	2 (17)	0 (0)
Race, *n* (%)				
Asian	7 (21)	4 (31)	2 (17)	1 (11)
White or Caucasian	21 (62)	6 (46)	8 (67)	7 (78)
Prefer not to answer	6 (18)	3 (23)	2 (17)	1 (11)
Ethnicity, *n* (%)				
Hispanic, Latino, or Spanish origin	1 (3)	1 (8)	0 (0)	0 (0)
Not Hispanic, Latino, or Spanish origin	28 (82)	8 (62)	11 (92)	9 (100)
Prefer not to answer	5 (15)	4 (31)	1 (8)	0 (0)
Practice setting, *n* (%)				
Private medical practice	10 (29)	3 (23)	3 (25)	4 (44)
Specialty group practice	5 (15)	3 (23)	1 (8)	1 (11)
Multi-specialty group practice	5 (15)	3 (23)	1 (8)	1 (11)
University hospital or university affiliated clinic	13 (38)	3 (23)	7 (58)	3 (33)
Hospital or clinic not associated with a university	1 (3)	1 (8)	0 (0)	0 (0)
Percentage of time providing direct patient care, *n* (%)				
50–74%	1 (3)	0 (0)	1 (8)	0 (0)
75% or more	33 (97)	13 (100)	11 (92)	9 (100)
Years practicing neurology, *n* (%)				
5–10 years	4 (12)	2 (15)	1 (8)	1 (11)
11–15 years	5 (15)	2 (15)	2 (17)	1 (11)
16–20 years	9 (26)	5 (38)	3 (25)	1 (11)
More than 20 years	16 (47)	4 (31)	6 (50)	6 (67)
Sub-specialty certifications*, *n* (%)				
Clinical neurophysiology	3 (9)	2 (15)	0 (0)	1 (11)
Epilepsy	1 (3)	0 (0)	0 (0)	1 (11)
Neurocritical care	1 (3)	1 (8)	0 (0)	0 (0)
Neurodevelopmental disabilities	1 (3)	0 (0)	0 (0)	1 (11)
Neuroimmunology and multiple sclerosis	2 (6)	0 (0)	2 (17)	0 (0)
Neuromuscular medicine	3 (9)	0 (0)	1 (8)	2 (22)
Patients encountered in the past 12 months with suspected FA, *n* (%)				
0 patients	18 (53)	3 (23)	8 (67)	7 (78)
1 to 5 patients	13 (38)	7 (54)	4 (33)	2 (22)
6 to 10 patients	2 (6)	2 (15)	0 (0)	0 (0)
10 + patients	1 (3)	1 (8)	0 (0)	0 (0)
Patients encountered in the past 12 months with confirmed FA, *n* (%)				
0 patients	23 (68)	2 (15)	12 (100)	9 (100)
1 to 5 patients	10 (29)	10 (77)	0 (0)	0 (0)
6 to 10 patients	0 (0)	0 (0)	0 (0)	0 (0)
10 + patients	1 (3)	1 (8)	0 (0)	0 (0)
Patients personally diagnosed with FA in the past 12 months, *n* (%)				
0 patients	23 (68)	3 (23)	11 (92)	9 (100)
1 to 5 patients	10 (29)	9 (69)	1 (8)	0 (0)
6 to 10 patients	0 (0)	0 (0)	0 (0)	0 (0)
10 + patients	1 (3)	1 (8)	0 (0)	0 (0)
Patients personally diagnosed with FA ever, *n* (%)				
0 patients	8 (24)	0 (0)	1 (8)	7 (78)
1 to 5 patients	19 (56)	6 (46)	11 (92)	2 (22)
6 to 10 patients	1 (3)	1 (8)	0 (0)	0 (0)
10 + patients	6 (18)	6 (46)	0 (0)	0 (0)

* All participants’ primary specialty was required to be neurology to be included in the study. Participants could select more than one sub-specialty certification. FA, Friedreich ataxia; SD, standard deviation.

**TABLE 2:** Consensus statements for likelihood questions after Round 3: full panel (*N* = 34).

Likelihood statement	Options rated	Median	IQR	Consensus reached?*
Section 1: Identification, diagnosis, management, assessment, and treatment of patients with FA				
Likelihood to use genetic testing to diagnose a patient who you suspect may have FA	N/A	100	5	Yes, Highly Likely
Likelihood to use genetic tests to diagnose a patient with suspected FA	Ataxia NextGen sequencing panel	25	40	No
	Ataxia panel with sequencing and triplet repeat analysis	75	44	No
	Frataxin sequencing	28	40	No
	Frataxin triplet repeat analysis	55	70	No
	Hereditary neuropathy panel	23	54	No
	Whole-exome sequencing	10	30	No
	Whole-genome sequencing ataxia panel	20	48	No
Likelihood to use tests for a patient who you suspect may have FA before diagnosis is genetically confirmed	Brain Magnetic Resonance Imaging (MRI)	100	10	Yes, Highly Likely
	Echocardiogram (cardiac echo)	18	50	No
	Electrocardiogram (EKG)	24	64	No
	Electromyogram (EMG)	75	44	No
	Glucose screening	63	99	No
	Nerve conduction velocity	75	54	No
	Neurological exam	100	0	Yes, Highly Likely
	Physical examination	100	0	Yes, Highly Likely
	Vitamin B12 test	98	33	No
	X-ray of head, spine and/or chest	3	28	No
Likelihood to use tests for a patient who you suspect may have FA after the diagnosis is genetically confirmed	Brain Magnetic Resonance Imaging (MRI)	55	88	No
	Echocardiogram (cardiac echo)	100	10	Yes, Highly Likely
	Electrocardiogram (EKG)	100	25	Yes, Highly Likely
	Electromyogram (EMG)	50	63	No
	Glucose screening	78	68	No
	Nerve conduction velocity	50	65	No
	Neurological exam	100	0	Yes, Highly Likely
	Physical examination	100	0	Yes, Highly Likely
	Vitamin B12 test	15	90	No
	X-ray of head, spine and/or chest	5	68	No
Likelihood to use methods to assess disease progression in patients diagnosed with FA	25-foot walk test	32	50	No
	Clinical outcome assessment scale	23	56	No
	General neurological exam	100	0	Yes, Highly Likely
	Quality of life measures	70	70	No
Likelihood to recommend pharmacological treatments for patients with FA	ACE inhibitors	0	20	Yes, Highly Unlikely
	Amantadine	8	24	Yes, Highly Unlikely
	Anti-arrhythmic agents	0	15	Yes, Highly Unlikely
	Baclofen	31	30	No
	Beta blockers	0	24	Yes, Highly Unlikely
	Coenzyme Q10/ Ubiquinol	8	58	No
	Diuretics	0	10	Yes, Highly Unlikely
	Gabapentin	50	30	No
	Idebenone	0	5	Yes, Highly Unlikely
	Omaveloxolone	40	88	No
	Riluzole	0	9	Yes, Highly Unlikely
Likelihood to refer a patient with FA to a specialist for specialized care	Cardiologist	100	18	Yes, Highly Likely
	Developmental pediatrician	10	36	No
	Endocrinologist	50	50	No
	Gastroenterologist	35	40	No
	Geneticist	80	49	No
	Hepatologist	23	50	No
	Movement disorder specialist	33	70	No
	Neuromuscular specialist	33	73	No
	Occupational therapist	98	29	No
	Orthopedic specialist	50	54	No
	Physical therapist	100	10	Yes, Highly Likely
	Speech pathologist	80	43	No
Section 2: Key challenges and unmet needs in diagnosing and treating patients with FA				
Patients with FA may present with symptoms that overlap with other ataxic or neurologic conditions that create challenges to making a correct diagnosis	N/A	90	17	Yes, Highly Likely
Likelihood of ability to confirm a diagnosis of FA starting from the time a patient initially presents to a healthcare clinic with symptoms	0–2 months	10	10	Yes, Highly Unlikely
	3–5 months	30	30	No
	6–11 months	60	33	No
	1–2 years	78	50	No
	>2 years	90	30	No
Section 3: Familiarity and use of COAS to monitor and assess FA disease activity				
Level of familiarity with COAS	BARS	5	23	Yes, Highly Unlikely
	ICARS	6	20	Yes, Highly Unlikely
	mFARS	10	20	Yes, Highly Unlikely
	SARA	6	20	Yes, Highly Unlikely
Level of feasibility to implement COAS in routine clinical practice	BARS	50	44	No
	ICARS	35	33	No
	mFARS	33	30	No
	SARA	40	29	No
Likelihood of use COAS in routine clinical practice	BARS	30	56	No
	ICARS	20	28	No
	mFARS	28	40	No
	SARA	30	54	No
Likelihood to administer COAS in routine clinical practice	Only once upon initial visit/ diagnosis	20	58	No
	During the initial visit and every subsequent follow-up visit	25	40	No
	During the initial visit and every 6 months after	38	44	No
	During the initial visit and once a year after	50	56	No
	During the initial visit and once every 2 years after	25	48	No
Estimated length of time to administer COAS during routine clinical visit	BARS	17	19	N/A for estimations
	ICARS	25	9	N/A for estimations
	mFARS	20	15	N/A for estimations
	SARA	15	12	N/A for estimations
Estimated clinically meaningful change in COAS per year	mFARS (given that scores typically worsen by ~2.0 points per year)	1.3	0.6	N/A for estimations
	SARA (given that scores typically worsen by ~1.0 points per year)	0.7	0.3	N/A for estimations
Likelihood of impact of patient’s score on a COAS on treatment or management decisions for FA	At the time of the first assessment	50	35	No
	Over time as their FA progresses	65	30	No
Likelihood of impact on patient access to new treatment if payers required mFARS as part of their coverage criteria	Cause delays to patient access to the new treatment	40	40	No
	Cause limited patient access to the new treatment	25	40	No
	It would not impact patient access to the new treatment	50	54	No
Likelihood of impact on patient access to new treatment if payers required SARA as part of their coverage criteria	Cause delays to patient access to the new treatment	25	40	No
	Cause limited patient access to the new treatment	20	34	No
	It would not impact patient access to the new treatment	50	53	No

* Consensus was considered to be present if the IQR was ≤ 25 during Round 3. For items where consensus was reached, cells are highlighted green if the median was 75–100 indicating the statement was highly likely and red if median was 0–25 indicating the statement was highly unlikely. BARS, Brief Ataxia Rating Scale; COAS, clinical outcomes assessment scales; FA, Friedreich ataxia; ICARS, International Cooperative Ataxia Rating Scale; IQR, Interquartile range; mFARS, modified Friedreich Ataxia Rating Scale; N/A, not applicable; SARA, Scale for the Assessment and Rating of Ataxia.

**Table 3:** Consensus statements for ranking questions after Round 3: full panel (*N* = 34).

Ranking statement	Options rated	Times ranked	Mean	Ranked among top-2: n	Ranked among top-2: %	Consensus reached?*
Section 1: Identification, diagnosis, management, assessment, and treatment of patients with FA						
Top 5 initial signs and symptoms that most commonly lead you to suspect a patient may have FA	Atrial fibrillation	1	4.0	0	0%	Yes, Highly Non-influential
	Difficulty in walking	33	1.8	28	82%	Yes, Highly Influential
	Dysphagia	3	3.7	1	3%	Yes, Highly Non-influential
	Family history of cerebellar ataxia	19	3.5	3	9%	Yes, Highly Non-influential
	Frequent falls	26	3.6	4	12%	Yes, Highly Non-influential
	Hand dexterity problem	16	3.6	3	9%	Yes, Highly Non-influential
	Headaches	0	Not ranked	0	0%	Yes, Highly Non-influential
	Hearing impairment	1	5.0	0	0%	Yes, Highly Non-influential
	High and painful foot arch/pes cavus	6	4.2	1	3%	Yes, Highly Non-influential
	Hypertrophic cardiomyopathy	2	2.5	1	3%	Yes, Highly Non-influential
	Muscle atrophy	3	3.7	1	3%	Yes, Highly Non-influential
	Numbness/loss of sensation	10	4.4	0	0%	Yes, Highly Non-influential
	Poor balance	31	1.8	25	74%	No
	Scoliosis	4	4.0	0	0%	Yes, Highly Non-influential
	Slurred speech	9	3.4	1	3%	Yes, Highly Non-influential
	Tremors	4	3.3	0	0%	Yes, Highly Non-influential
	Vision impairment	1	5.0	0	0%	Yes, Highly Non-influential
	Young onset diabetes	1	5.0	0	0%	Yes, Highly Non-influential
Top 3 factors considered when making a treatment recommendation for a patient with FA	Efficacy of the treatment	32	1.2	30	88%	Yes, Highly Influential
	FA severity	6	2.2	5	15%	Yes, Highly Non-influential
	Insurance coverage/cost of treatment	6	2.8	1	3%	Yes, Highly Non-influential
	Patient functionality	10	2.3	7	21%	Yes, Highly Non-influential
	Patient preference	6	2.0	5	15%	Yes, Highly Non-influential
	Patient’s comorbidities	3	3.0	0	0%	Yes, Highly Non-influential
	Patient’s support system	0	Not ranked	0	0%	Yes, Highly Non-influential
	Personal/colleagues’ experience with the treatment	1	3.0	0	0%	Yes, Highly Non-influential
	Safety/adverse events associated with the treatment	22	2.5	11	32%	No
	Treatment guidelines/published literature	16	2.1	9	26%	No
Section 2: Key challenges and unmet needs in diagnosing and treating patients with FA						
Primary challenges in the FA diagnostic process	Access to/cost of diagnostic tests (e.g., genetic testing)	34	4.2	10	29%	No
	Atypical ataxia presentation	34	2.9	15	44%	No
	Delays from referrals to specialists	34	4.9	5	15%	Yes, Highly Non-influential
	Determining onset of the disease	34	5.2	2	6%	Yes, Highly Non-influential
	Early brain MRIs do not show cerebellar atrophy	34	4.6	5	15%	Yes, Highly Non-influential
	Presence of non-ataxia symptoms	34	3.9	9	26%	No
	Presentation of only mild symptoms	34	2.4	22	65%	No
Top 3 drivers of delayed diagnoses in FA	Delays due to genetic testing costs/lack of insurance coverage	25	1.9	20	59%	No
	Delays from misdiagnosis by either the primary care provider or specialist	30	1.7	22	65%	No
	Delays from referrals to specialists	25	2.2	14	41%	No
	Delays related to age of onset	6	2.2	4	12%	Yes, Highly Non-influential
	Delays related to presence of comorbidities	7	2.6	3	9%	Yes, Highly Non-influential
	Delays related to site (e.g., part of body) of disease onset	5	2.0	3	9%	Yes, Highly Non-influential
	The notion that there is nothing a doctor can do even with a diagnosis	4	2.3	2	6%	Yes, Highly Non-influential
Primary impacts of misdiagnosing a patient with another ataxia or neurologic condition when they actually have FA	Inappropriate treatment or interventions	34	2.6	14	41%	No
	Missing potential complications from cardiac or endocrinological manifestations	34	1.9	24	71%	No
	Missing the opportunity to delay disease progression	34	2.5	19	56%	No
	Unnecessary investigations and procedures related to the incorrect diagnosis (e.g., additional tests, imaging, etc.)	34	3.0	11	32%	No
Primary challenges related to the treatment of patients with FA	Availability of disease modifying treatment options	34	1.8	27	79%	Yes, Highly Influential
	Limited effectiveness of treatments to manage symptoms	34	2.4	25	74%	No
	Limited number of tertiary care centers and localized knowledge of the disease	34	3.6	7	21%	Yes, Highly Non-influential
	Management of comorbidities	34	4.2	3	9%	Yes, Highly Non-influential
	Patient adherence to treatment	34	5.2	2	6%	Yes, Highly Non-influential
	Patient’s missing follow-up visits	34	6.1	2	6%	Yes, Highly Non-influential
	Treatment-related side effects	34	4.6	2	6%	Yes, Highly Non-influential
Section 3: Familiarity and use of COAS to monitor and assess FA disease activity						
Most appropriate COAS to use for the assessment of FA disease activity and treatment effects in routine clinical practice	BARS	34	2.4	19	56%	No
	ICARS	34	2.8	13	38%	No
	mFARS	34	1.9	22	65%	No
	SARA	34	2.9	14	41%	No
Key challenges that limit adoption of the use of the mFARS in routine clinical practice to assess FA patients	Does not capture relevant components to fully assess clinical benefit or disease activity	34	4.3	2	6%	Yes, Highly Non-influential
	It is not a clinically meaningful outcome for the patient	34	3.9	3	9%	Yes, Highly Non-influential
	Lack of familiarity among physicians with the mFARS	34	1.8	27	79%	Yes, Highly Influential
	Takes too long to administer	34	2.1	25	74%	No
	There are other scales or methods to assess disease activity that are more practical for use in routine clinical practice	34	2.9	11	32%	No
Key challenges that limit adoption of the use of the SARA in routine clinical practice to assess FA patients	Does not capture relevant components to fully assess clinical benefit	34	3.5	6	18%	Yes, Highly Non-influential
	It is not a clinically meaningful outcome for the patient	34	3.6	6	18%	Yes, Highly Non-influential
	Lack of familiarity among physicians with the SARA	34	1.8	28	82%	Yes, Highly Influential
	Takes too long to administer	34	2.5	21	62%	No
	There are other scales or methods to assess disease activity that are more practical for use in routine clinical practice	34	3.6	7	21%	Yes, Highly Non-influential

* For items where consensus was reached, cells are highlighted green if the % ranked among top-2 was ≥ 75 indicating the option was highly influential and red if the % ranked among top-2 was ≤ 25 indicating the option was highly non-influential. BARS, Brief Ataxia Rating Scale; COAS, clinical outcomes assessment scales; FA, Friedreich ataxia; ICARS, International Cooperative Ataxia Rating Scale; mFARS, modified Friedreich Ataxia Rating Scale; MRI, magnetic resonance imaging; SARA, Scale for the Assessment and Rating of Ataxia.

### 3.2 Identification, diagnosis, and management of patients with FA

Difficulty in walking (mean rank = 1.8, ranked top 2: 82%) and poor balance (mean rank = 1.8, ranked top 2: 74%) are initial symptoms or signs most commonly associated with suspected FA. General neurological exam was the most frequently used method to assess disease progression in patients diagnosed with FA (median = 100, IQR = 0). Patients with FA are most likely to be referred to a physical therapist (median = 100, IQR = 10) and a cardiologist (median = 100, IQR = 18) for specialized care.

### 3.3 Key challenges and unmet needs healthcare providers face in diagnosing and treating patients with FA

Participants strongly agreed patients with FA are highly likely to present with symptoms that overlap with other ataxic or neurologic conditions, which hinders making a correct and timely diagnosis (median = 90, IQR = 17). Diagnosing patients is challenging due to mild symptoms at onset (mean rank = 2.4, ranked top 2: 65%) and atypical ataxia presentation (mean rank = 2.9, ranked top 2: 44%) as well as overlap with other ataxic or neurologic conditions (median = 90, IQR = 17). Furthermore, misdiagnosis was perceived as the most important driver in delayed diagnoses in FA (mean rank = 1.7, ranked top 2: 65%).

Delayed diagnosis might lead to missing potential complications from cardiac or endocrinological manifestations (mean rank = 1.9, ranked top 2: 71%) and missing the opportunity to delay disease progression (mean rank = 2.5, ranked top 2: 56%). Availability of disease modifying treatment options (mean rank = 1.8, ranked top 2: 79%) and limited effectiveness of treatments to manage symptoms (mean rank = 2.4, ranked top 2: 74%) were the most important challenges in treating patients with FA.

### 3.4 Familiarity and use of COAS to monitor and assess FA disease activity

Neurologists were highly unfamiliar with any of the COAS provided (median range: 5–10, IQR range: 20–23), and were unlikely to use any of the COAS in routine clinical practice (median range: 20–30, IQR range: 28–56; Figure 1). Further, none of the COAS were deemed highly feasible to implement in routine clinical practice (median range: 33–50, IQR range: 29–44). Participants estimated they could complete each COAS in 20–30 min on average, with responses ranging from 5 min to 2 h. Lack of familiarity and length of administration time were reported as the key challenges limiting adoption of known ataxia rating scales, specifically, both the mFARS (mean rank = 1.8, ranked top 2: 79%) and the SARA (mean rank = 1.8, ranked top 2: 82%), in routine clinical practice to assess FA patients.

**Figure 1:**
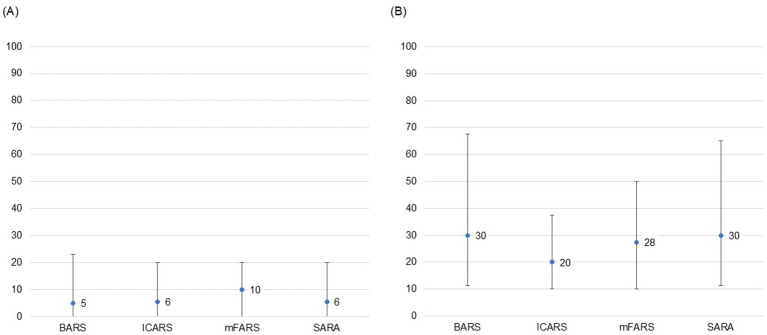
Participant level of familiarity and likelihood of use of COAS in routine clinical practice. **(A)** Level of familiarity with COAS. **(B)** Likelihood of use of COAS in routine clinical practice. 0 = highly unlikely; 100 = highly likely. Point estimates represent medians and bars represent IQRs based on responses during Round 3. **(A)** Participants strongly agreed that they were highly unfamiliar with any of the COAS provided. **(B)** Participants were unlikely to use ICARS and moderately unlikely to use the other scales in their clinical practice but did not reach consensus around any of the options provided. BARS, Brief Ataxia Rating Scale; COAS, clinical outcomes assessment scales; ICARS, International Cooperative Ataxia Rating Scale; IQR, interquartile range; mFARS, modified Friedreich Ataxia Rating Scale; SARA, Scale for the Assessment and Rating of Ataxia.

### 3.5 Differences between experienced FA-treaters and the overall sample of US-neurologists

Consensus statement results for likelihood and ranking questions among the substantial/recent FA experience subgroup are presented in Supplementary Tables 1, 2. Experienced FA-treaters exhibited consensus regarding both poor balance and difficulty walking as initial symptoms or signs most commonly associated with suspected FA, while the overall sample only achieved consensus around difficulty walking. Experienced FA-treaters strongly agreed they were highly likely to use frataxin triple repeat analysis, while the overall sample was unlikely to use any particular genetic test.

Both experienced FA-treaters and the overall sample were most likely to use a general neurological exam to assess disease progression in a patient with FA, but experienced FA-treaters emphasized they were unlikely to use the 25-foot walk test, though with no consensus. Experienced FA-treaters were highly unlikely to use all clinical COAS in their clinical practice. The highest estimate for completion of the COAS provided by an experienced FA-treater was 45 min, compared to a maximum of 2 h for the overall sample.

## 4 Discussion

FA is a rare genetic neurological disease requiring a broad multidisciplinary approach to manage symptoms. While the disease has been known since 1863 in the medical community, until recently, no disease-modifying treatments were available. Little is known about the level of familiarity neurologists have with this disease, as well as management and treatment strategies employed. Given the recent approval of omaveloxolone as a treatment for FA, there is an urgent need to understand how neurologists recognize, diagnose, assess, manage and treat patients with FA in their clinical practice (23). This study systematically elicited opinions on these questions from a panel of neurologists with varying prior exposure to patients with FA.

Specifically, we used the Delphi technique to identify areas of agreement (consensus) and disagreement between neurologists who are most likely to diagnose and treat patients with FA, given their neurology specialization. We found that overall, neurologists exhibit consensus in only a few areas pertaining to management and assessment of patients with FA. This indicates that there is heterogeneity in patient care and management, that is not necessarily due to patient characteristics or FA disease stage but potentially due to provider practices, preferences, as well as disease-specific awareness and education. It is possible, given the recent approval of omaveloxolone, that patient management and provider preferences may become more homogeneous, however it will take time to evolve.

Areas with the highest level of consensus included lack of familiarity with COAS, access to and familiarity with specific tests used to confirm FA diagnoses, as well as the availability of and familiarity with disease-modifying therapies for patients with FA. Neurologists on our panel were most likely to use a general neurological exam to monitor and assess disease progression in patients with FA, and emphasized the COAS most commonly used to assess FA patients in clinical trial settings would likely be too time consuming and resource-intensive to implement in routine clinical practice. These COAS are valuable but should not be fundamental in their current state in the assessment and treatment pathway for a patient with FA.

Lastly, neurologists also stressed challenges in the diagnostic process, ranking mild symptoms and overlapping symptom burden as the primary reasons for delays in diagnosis. Although overlapping symptoms with other neurological diseases will continue to remain a challenge in the FA diagnostic process, increased education and awareness among neurologists could support earlier detection and diagnosis. Specifically, it is important to educate health care professionals regarding differential diagnoses in ataxia, appropriate genetic tests, and timely referrals to reduce challenges in diagnostic efforts and shorten time to diagnosis for patients. As new disease-modifying treatments for FA emerge, earlier diagnosis of FA is essential to support early intervention and thus improve patient outcomes by slowing disease progression and enhancing patient quality of life.

### 4.1 Study strengths and limitations

Our study design has several strengths. Principally, this is the first study we are aware of that convenes a panel of neurologists to obtain a broader understanding of FA decision-making practices, particularly opinions around the use of COAS for monitoring patients with FA. The use of the Delphi method as a structured expert elicitation technique enables the capturing of perspectives and opinions across a broad spectrum of participants. The inclusion of neurologists who have experience with FA and those who have not encountered a patient with FA previously but may in the future allows us to gain a better understanding of real-world clinical practices across neurologists who have varying experiences and familiarity with FA. This methodology facilitates an in-depth exploration of attitudes and opinions that is not possible in quantitative surveys.

Aside from its strengths, the study design also has limitations that merit discussion. The modified Delphi method typically relies on a small sample of participants, made up of experts in the field, and thus may not be generalizable beyond the community surveyed. In particular, patients with FA may be seen by a wide range of health care providers beyond neurologists, such as general practitioners, pediatricians, nurse practitioners or physician assistants. However, this study assumes that based on their training and specialization, neurologists tend to be most likely to encounter patients with FA and be the main decision-maker for clinical management. While study results cannot be viewed as representative of the overall neurologist population, the Delphi methodology aims to systematically aggregate subject matter expert estimates around key questions of uncertainty in the absence of long-term empirical evidence. Given participation was voluntary, self-selection bias may be present. Participants involved in this study may also be more enthusiastic about the study subject than their peers who elect not to participate. Nevertheless, we anticipate that the study results provide unique views and valuable insights into key issues regarding the management and treatment of patients with FA.

### 4.2 Considerations for future research

It would be valuable to repeat this Delphi study in future years to understand the evolution in approaches to management and assessment of patients with FA as new treatment options become available. Similarly, a Delphi executed in non-US countries to understand global management and assessment practices would be valuable in the evolution of care for patients with FA worldwide. As patients with FA are ideally under the care of a multidisciplinary team, broader specialty outreach would allow for a comprehensive representation of the types of clinicians managing FA patients in real-world settings and elucidate general disease management and treatment approaches. Additionally, challenges in recognizing, diagnosing and evaluating disease progression by multidisciplinary team members across specialties would be important to understand to optimize patient care. Future research using real-world observational data or patient registries can complement expert consensus findings and provide a more comprehensive understanding of real-world clinical management of FA across various clinical settings.

Further, this Delphi highlights challenges in using current COAS in everyday clinical practice and findings could support future discussion around these tools. As COAS are typically used in clinical trial settings, there are different, unique challenges in utilizing them in real-world routine clinical practice due to time and resource constraints. Insights from this study demonstrate the importance of thoughtfully selecting clinical trial measures with input from a variety of providers across care settings to ensure greater alignment with real-world clinical workflows and ultimately support more efficient care delivery for patients.

In conclusion, this Delphi study provides valuable insights and opinions from a panel of US neurologists around key questions in management and assessment of patients with FA. Neurologists were only able to reach consensus on a small portion of questions regarding FA diagnosis and assessment, perhaps due to the rarity of disease and panelists’ varying FA experience. To improve and standardize treatment and management processes for patients with FA, it is important to establish best practices and educate potential FA treaters as new therapies emerge. Furthermore, to address existing gaps in expertise and consistency of care, establishing multidisciplinary, centralized care hubs can help standardize clinical management, improve the diagnostic process, and support ongoing research for this complex, rare disease.

## Data Availability

The raw data supporting the conclusions of this article will be made available by the authors, without undue reservation.
