# Supplementary material for: Delphi study to elicit expert consensus around decision-making in the treatment of Friedreich ataxia
Source: Front Neurol. 2025 Oct 24;16:1669059. doi: 10.3389/fneur.2025.1669059 (PMC12591944; doi:10.3389/fneur.2025.1669059)
Supplement: Supplementary file 1 [file Supplementary_file_1.docx]

Supplementary Material

Supplementary Table 1. Consensus statements for likelihood questions after Round 3: Substantial/recent experience subgroup (N=13)

| **Likelihood statement** | **Options rated** | **Median** | **IQR** | **Consensus reached?*** |
| --- | --- | --- | --- | --- |
| **Section 1: Identification, diagnosis, management, assessment, and treatment of patients with FA** | | | | |
| Likelihood to use genetic testing to diagnose a patient who you suspect may have FA | N/A | 100 | 5 | Yes, Highly Likely |
| Likelihood to use genetic tests to diagnose a patient with suspected FA | Ataxia NextGen sequencing panel | 30 | 50 | No |
|  | Ataxia panel with sequencing and triplet repeat analysis | 75 | 40 | No |
|  | Frataxin sequencing | 30 | 50 | No |
|  | Frataxin triplet repeat analysis | 80 | 80 | No |
|  | Hereditary neuropathy panel | 30 | 60 | No |
|  | Whole-exome sequencing | 15 | 30 | No |
|  | Whole-genome sequencing ataxia panel | 20 | 50 | No |
| Likelihood to use tests for a patient who you suspect may have FA **before** diagnosis is genetically confirmed | Brain Magnetic Resonance Imaging (MRI) | 95 | 20 | Yes, Highly Likely |
|  | Echocardiogram (cardiac echo) | 20 | 30 | No |
|  | Electrocardiogram (EKG) | 22 | 75 | No |
|  | Electromyogram (EMG) | 75 | 30 | No |
|  | Glucose screening | 37 | 90 | No |
|  | Nerve conduction velocity | 80 | 40 | No |
|  | Neurological exam | 100 | 0 | Yes, Highly Likely |
|  | Physical examination | 100 | 0 | Yes, Highly Likely |
|  | Vitamin B12 test | 95 | 40 | No |
|  | X-ray of head, spine and/or chest | 10 | 20 | Yes, Highly Unlikely |
| Likelihood to use tests for a patient who you suspect may have FA **after** the diagnosis is genetically confirmed | Brain Magnetic Resonance Imaging (MRI) | 50 | 80 | No |
|  | Echocardiogram (cardiac echo) | 95 | 20 | Yes, Highly Likely |
|  | Electrocardiogram (EKG) | 90 | 25 | Yes, Highly Likely |
|  | Electromyogram (EMG) | 25 | 75 | No |
|  | Glucose screening | 75 | 63 | No |
|  | Nerve conduction velocity | 45 | 80 | No |
|  | Neurological exam | 100 | 10 | Yes, Highly Likely |
|  | Physical examination | 100 | 10 | Yes, Highly Likely |
|  | Vitamin B12 test | 20 | 95 | No |
|  | X-ray of head, spine and/or chest | 10 | 60 | No |
| Likelihood to use methods to assess disease progression in patients diagnosed with FA | 25-foot walk test | 25 | 50 | No |
|  | Clinical outcome assessment scale | 40 | 50 | No |
|  | General neurological exam | 100 | 15 | Yes, Highly Likely |
|  | Quality of life measures | 70 | 70 | No |
| Likelihood to recommend pharmacological treatments for patients with FA | ACE inhibitors | 5 | 32 | No |
|  | Amantadine | 10 | 20 | Yes, Highly Unlikely |
|  | Anti-arrhythmic agents | 10 | 30 | No |
|  | Baclofen | 30 | 15 | Yes, Moderately Unlikely |
|  | Beta blockers | 10 | 30 | No |
|  | Coenzyme Q10/ Ubiquinol | 10 | 50 | No |
|  | Diuretics | 0 | 10 | Yes, Highly Unlikely |
|  | Gabapentin | 50 | 30 | No |
|  | Idebenone | 0 | 5 | Yes, Highly Unlikely |
|  | Omaveloxolone | 50 | 80 | No |
|  | Riluzole | 0 | 5 | Yes, Highly Unlikely |
| Likelihood to refer a patient with FA to a specialist for specialized care | Cardiologist | 90 | 55 | No |
|  | Developmental pediatrician | 0 | 40 | No |
|  | Endocrinologist | 30 | 30 | No |
|  | Gastroenterologist | 25 | 44 | No |
|  | Geneticist | 75 | 40 | No |
|  | Hepatologist | 1 | 40 | No |
|  | Movement disorder specialist | 30 | 60 | No |
|  | Neuromuscular specialist | 5 | 60 | No |
|  | Occupational therapist | 90 | 40 | No |
|  | Orthopedic specialist | 50 | 70 | No |
|  | Physical therapist | 100 | 25 | Yes, Highly Likely |
|  | Speech pathologist | 80 | 40 | No |
| **Section 2: Key challenges and unmet needs in diagnosing and treating patients with FA** | | | | |
| Patients with FA may present with symptoms that overlap with other ataxic or neurologic conditions that create challenges to making a correct diagnosis | N/A | 90 | 18 | Yes, Highly Likely |
| Likelihood of ability to confirm a diagnosis of FA starting from the time a patient initially presents to a healthcare clinic with symptoms | 0 – 2 months | 20 | 10 | Yes, Highly Unlikely |
|  | 3 – 5 months | 30 | 35 | No |
|  | 6 – 11 months | 55 | 35 | No |
|  | 1 – 2 years | 85 | 40 | No |
|  | >2 years | 95 | 30 | No |
| **Section 3: Familiarity and use of COAS to monitor and assess FA disease activity** | | | | |
| Level of familiarity with COAS | BARS | 20 | 60 | No |
|  | ICARS | 20 | 50 | No |
|  | mFARS | 20 | 70 | No |
|  | SARA | 20 | 45 | No |
| Level of feasibility to implement COAS in routine clinical practice | BARS | 50 | 45 | No |
|  | ICARS | 40 | 20 | Yes, Moderately Unlikely |
|  | mFARS | 35 | 30 | No |
|  | SARA | 30 | 25 | Yes, Moderately Unlikely |
| Likelihood of use COAS in routine clinical practice | BARS | 25 | 45 | No |
|  | ICARS | 20 | 20 | Yes, Highly Unlikely |
|  | mFARS | 25 | 35 | No |
|  | SARA | 25 | 20 | Yes, Highly Unlikely |
| Likelihood to administer COAS in routine clinical practice | Only once upon initial visit/ diagnosis | 20 | 65 | No |
|  | During the initial visit and every subsequent follow-up visit | 20 | 20 | Yes, Highly Unlikely |
|  | During the initial visit and every 6 months after | 50 | 30 | No |
|  | During the initial visit and once a year after | 60 | 30 | No |
|  | During the initial visit and once every 2 years after | 40 | 45 | No |
| Estimated length of time to administer COAS during routine clinical visit | BARS | 15 | 15 | N/A for estimations |
|  | ICARS | 25 | 10 | N/A for estimations |
|  | mFARS | 20 | 15 | N/A for estimations |
|  | SARA | 15 | 10 | N/A for estimations |
| Estimated clinically meaningful change in COAS per year | mFARS (given that scores typically worsen by ~2.0 points per year) | 1.4 | 0.4 | N/A for estimations |
|  | SARA (given that scores typically worsen by ~1.0 points per year) | 0.7 | 0.2 | N/A for estimations |
| Likelihood of impact of patient's score on a COAS on treatment or management decisions for FA | At the time of the first assessment | 50 | 15 | Yes, Moderately Likely |
|  | Over time as their FA progresses | 65 | 30 | No |
| Likelihood of impact on patient access to new treatment if payers required **mFARS** as part of their coverage criteria | Cause delays to patient access to the new treatment | 40 | 40 | No |
|  | Cause limited patient access to the new treatment | 20 | 40 | No |
|  | It would not impact patient access to the new treatment | 50 | 50 | No |
| Likelihood of impact on patient access to new treatment if payers required **SARA** as part of their coverage criteria | Cause delays to patient access to the new treatment | 20 | 20 | Yes, Highly Unlikely |
|  | Cause limited patient access to the new treatment | 20 | 30 | No |
|  | It would not impact patient access to the new treatment | 50 | 70 | No |

*Consensus was considered to be present if the IQR was ≤ 25 during Round 3. For items where consensus was reached, cells are highlighted green if the median was 75-100 indicating the statement was highly likely, yellow if median was 26-74 indicating the statement was moderately likely, and red if median was 0-25 indicating the statement was highly unlikely.

BARS: Brief Ataxia Rating Scale, COAS: clinical outcomes assessment scales, FA: Friedreich ataxia, ICARS: International Cooperative Ataxia Rating Scale, IQR: Interquartile range, mFARS: modified Friedreich Ataxia Rating Scale, N/A: not applicable, SARA: Scale for the Assessment and Rating of Ataxia.

Supplementary Table 2. Consensus statements for ranking questions after Round 3: Substantial/recent experience subgroup (N=13)

| **Ranking statement** | **Options rated** | **Times ranked** | **Mean** | **Ranked among top-2: n** | **Ranked among top-2: %** | **Consensus reached?*** |
| --- | --- | --- | --- | --- | --- | --- |
| **Section 1: Identification, diagnosis, management, assessment, and treatment of patients with FA** | | | | | | |
| Top 5 initial signs and symptoms that most commonly lead you to suspect a patient may have FA | Atrial fibrillation | 0 | Not ranked | 0 | 0% | Yes, Highly Non-influential |
|  | Difficulty in walking | 13 | 1.6 | 12 | 92% | Yes, Highly Influential |
|  | Dysphagia | 1 | 4.0 | 0 | 0% | Yes, Highly Non-influential |
|  | Family history of cerebellar ataxia | 6 | 3.3 | 1 | 8% | Yes, Highly Non-influential |
|  | Frequent falls | 13 | 3.5 | 2 | 15% | Yes, Highly Non-influential |
|  | Hand dexterity problem | 9 | 4.0 | 0 | 0% | Yes, Highly Non-influential |
|  | Headaches | 0 | Not ranked | 0 | 0% | Yes, Highly Non-influential |
|  | Hearing impairment | 0 | Not ranked | 0 | 0% | Yes, Highly Non-influential |
|  | High and painful foot arch/pes cavus | 1 | 4.0 | 0 | 0% | Yes, Highly Non-influential |
|  | Hypertrophic cardiomyopathy | 0 | Not ranked | 0 | 0% | Yes, Highly Non-influential |
|  | Muscle atrophy | 1 | 5.0 | 0 | 0% | Yes, Highly Non-influential |
|  | Numbness/loss of sensation | 4 | 4.5 | 0 | 0% | Yes, Highly Non-influential |
|  | Poor balance | 12 | 1.7 | 10 | 77% | Yes, Highly Influential |
|  | Scoliosis | 1 | 5.0 | 0 | 0% | Yes, Highly Non-influential |
|  | Slurred speech | 3 | 3.7 | 1 | 8% | Yes, Highly Non-influential |
|  | Tremors | 0 | Not ranked | 0 | 0% | Yes, Highly Non-influential |
|  | Vision impairment | 1 | 5.0 | 0 | 0% | Yes, Highly Non-influential |
|  | Young onset diabetes | 0 | Not ranked | 0 | 0% | Yes, Highly Non-influential |
| Top 3 factors considered when making a treatment recommendation for a patient with FA | Efficacy of the treatment | 13 | 1.1 | 13 | 100% | Yes, Highly Influential |
|  | FA severity | 2 | 2.5 | 1 | 8% | Yes, Highly Non-influential |
|  | Insurance coverage/cost of treatment | 0 | Not ranked | 0 | 0% | Yes, Highly Non-influential |
|  | Patient functionality | 4 | 2.3 | 3 | 23% | Yes, Highly Non-influential |
|  | Patient preference | 4 | 2.3 | 3 | 23% | Yes, Highly Non-influential |
|  | Patient’s comorbidities | 2 | 3.0 | 0 | 0% | Yes, Highly Non-influential |
|  | Patient’s support system | 0 | Not ranked | 0 | 0% | Yes, Highly Non-influential |
|  | Personal/colleagues’ experience with the treatment | 0 | Not ranked | 0 | 0% | Yes, Highly Non-influential |
|  | Safety/adverse events associated with the treatment | 10 | 2.4 | 5 | 38% | No |
|  | Treatment guidelines/published literature | 4 | 2.8 | 1 | 8% | Yes, Highly Non-influential |
| **Section 2: Key challenges and unmet needs in diagnosing and treating patients with FA** | | | | | | |
| Primary challenges in the FA diagnostic process | Access to/cost of diagnostic tests (e.g., genetic testing) | 13 | 5.6 | 1 | 8% | Yes, Highly Non-influential |
|  | Atypical ataxia presentation | 13 | 2.8 | 6 | 46% | No |
|  | Delays from referrals to specialists | 13 | 4.0 | 4 | 31% | No |
|  | Determining onset of the disease | 13 | 4.7 | 1 | 8% | Yes, Highly Non-influential |
|  | Early brain MRIs do not show cerebellar atrophy | 13 | 4.8 | 2 | 15% | Yes, Highly Non-influential |
|  | Presence of non-ataxia symptoms | 13 | 3.4 | 4 | 31% | No |
|  | Presentation of only mild symptoms | 13 | 2.6 | 8 | 62% | No |
| Top 3 drivers of delayed diagnoses in FA | Delays due to genetic testing costs/lack of insurance coverage | 6 | 2.5 | 3 | 23% | Yes, Highly Non-influential |
|  | Delays from misdiagnosis by either the primary care provider or specialist | 13 | 1.7 | 10 | 77% | Yes, Highly Influential |
|  | Delays from referrals to specialists | 10 | 2.2 | 5 | 38% | No |
|  | Delays related to age of onset | 4 | 2.0 | 3 | 23% | Yes, Highly Non-influential |
|  | Delays related to presence of comorbidities | 1 | 2.0 | 1 | 8% | Yes, Highly Non-influential |
|  | Delays related to site (e.g., part of body) of disease onset | 2 | 1.5 | 2 | 15% | Yes, Highly Non-influential |
|  | The notion that there is nothing a doctor can do even with a diagnosis | 3 | 2.0 | 2 | 15% | Yes, Highly Non-influential |
| Primary impacts of misdiagnosing a patient with another ataxia or neurologic condition when they actually have FA | Inappropriate treatment or interventions | 13 | 2.5 | 6 | 46% | No |
|  | Missing potential complications from cardiac or endocrinological manifestations | 13 | 2.2 | 9 | 69% | No |
|  | Missing the opportunity to delay disease progression | 13 | 2.2 | 8 | 62% | No |
|  | Unnecessary investigations and procedures related to the incorrect diagnosis (e.g., additional tests, imaging, etc.) | 13 | 3.2 | 3 | 23% | Yes, Highly Non-influential |
| Primary challenges related to the treatment of patients with FA | Availability of disease modifying treatment options | 13 | 1.7 | 11 | 85% | Yes, Highly Influential |
|  | Limited effectiveness of treatments to manage symptoms | 13 | 2.4 | 10 | 77% | Yes, Highly Influential |
|  | Limited number of tertiary care centers and localized knowledge of the disease | 13 | 3.8 | 2 | 15% | Yes, Highly Non-influential |
|  | Management of comorbidities | 13 | 4.4 | 1 | 8% | Yes, Highly Non-influential |
|  | Patient adherence to treatment | 13 | 5.5 | 0 | 0% | Yes, Highly Non-influential |
|  | Patient’s missing follow-up visits | 13 | 5.8 | 1 | 8% | Yes, Highly Non-influential |
|  | Treatment-related side effects | 13 | 4.4 | 1 | 8% | Yes, Highly Non-influential |
| **Section 3: Familiarity and use of COAS to monitor and assess FA disease activity** | | | | | | |
| Most appropriate COAS to use for the assessment of FA disease activity and treatment effects in routine clinical practice | BARS | 13 | 2.4 | 8 | 62% | No |
|  | ICARS | 13 | 3.0 | 4 | 31% | No |
|  | mFARS | 13 | 1.7 | 9 | 69% | No |
|  | SARA | 13 | 2.9 | 5 | 38% | No |
| Key challenges that limit adoption of the use of the **mFARS** in routine clinical practice to assess FA patients | Doesn’t capture relevant components to fully assess clinical benefit or disease activity | 13 | 3.8 | 2 | 15% | Yes, Highly Non-influential |
|  | It is not a clinically meaningful outcome for the patient | 13 | 4.2 | 1 | 8% | Yes, Highly Non-influential |
|  | Lack of familiarity among physicians with the mFARS | 13 | 2.4 | 7 | 54% | No |
|  | Takes too long to administer | 13 | 1.9 | 10 | 77% | Yes, Highly Influential |
|  | There are other scales or methods to assess disease activity that are more practical for use in routine clinical practice | 13 | 2.8 | 6 | 46% | No |
| Key challenges that limit adoption of the use of the SARA in routine clinical practice to assess FA patients | Doesn’t capture relevant components to fully assess clinical benefit | 13 | 2.9 | 5 | 38% | No |
|  | It is not a clinically meaningful outcome for the patient | 13 | 3.3 | 3 | 23% | Yes, Highly Non-influential |
|  | Lack of familiarity among physicians with the SARA | 13 | 2.6 | 8 | 62% | No |
|  | Takes too long to administer | 13 | 2.5 | 8 | 62% | No |
|  | There are other scales or methods to assess disease activity that are more practical for use in routine clinical practice | 13 | 3.6 | 2 | 15% | Yes, Highly Non-influential |

* For items where consensus was reached, cells are highlighted green if the % ranked among top-2 was ≥ 75 indicating the option was highly influential and red if the % ranked among top-2 was ≤ 25 indicating the option was highly non-influential.

BARS: Brief Ataxia Rating Scale, COAS: clinical outcomes assessment scales, FA: Friedreich ataxia, ICARS: International Cooperative Ataxia Rating Scale, mFARS: modified Friedreich Ataxia Rating Scale, MRI: magnetic resonance imaging, SARA: Scale for the Assessment and Rating of Ataxia.
